# Chemical constituents and antihistamine activity of *Bixa orellana* leaf extract

**DOI:** 10.1186/1472-6882-13-32

**Published:** 2013-02-14

**Authors:** Yoke Keong Yong, Zainul Amiruddin Zakaria, Arifah Abdul Kadir, Muhammad Nazrul Somchit, Gwendoline Ee Cheng Lian, Zuraini Ahmad

**Affiliations:** 1 Department of Biomedical Sciences, Faculty of Medicine and Health Sciences, Universiti Putra Malaysia, 43400 UPM Serdang, Selangor, Malaysia; 2 Department of Preclinical Science, Faculty of Veterinary Medicine, Universiti Putra Malaysia, 43400 UPM Serdang, Selangor, Malaysia; 3 Department of Chemistry, Faculty of Science, Universiti Putra Malaysia, 43400 UPM Serdang, Selangor, Malaysia

**Keywords:** Bixa orellana, Histamine, Anti-inflammatory, Vascular permeability

## Abstract

**Background:**

*Bixa orellana L.* has been traditionally used in Central and South America to treat a number of ailments, including internal inflammation, and in other tropical countries like Malaysia as treatment for gastric ulcers and stomach discomfort. The current study aimed to determine the major chemical constituents of the aqueous extract of *B. orellana* (AEBO) and to evaluate the antihistamine activity of AEBO during acute inflammation induced in rats.

**Methods:**

Acute inflammation was produced by subplantar injection of 0.1 mL of 0.1% histamine into the right hind paw of each rat in the control and treatment groups. The degree of edema was measured before injection and at the time points of 30, 60, 120, 180, 240 and 300 min after injection. Changes of peritoneal vascular permeability were studied using Evans blue dye as a detector. Vascular permeability was evaluated by the amount of dye leakage into the peritoneal cavity in rats. To evaluate the inhibitory effect of AEBO on biochemical mediators of vascular permeability, the levels of nitric oxide (NO) and vascular endothelial growth factor (VEGF) were determined in histamine-treated paw tissues. The major constituents of AEBO were determined by gas chromatography–mass spectrometry (GC-MS) analysis.

**Results:**

AEBO produced a significant inhibition of histamine-induced paw edema starting at 60 min time point, with maximal percentage of inhibition (60.25%) achieved with a dose of 150 mg/kg of AEBO at 60 min time point. Up to 99% of increased peritoneal vascular permeability produced by histamine was successfully suppressed by AEBO. The expression of biochemical mediators of vascular permeability, NO and VEGF, was also found to be downregulated in the AEBO treated group. Gas chromatography–mass spectrometry (GC-MS) analysis revealed that the major constituent in AEBO was acetic acid.

**Conclusions:**

The experimental findings demonstrated that the anti-inflammatory activity of AEBO was due to its inhibitory effect on vascular permeability, which was suppressed as a result of the reduced expression of biochemical mediators (NO and VEGF) in tissues. Our results contribute towards the validation of the traditional use of *Bixa orellana* in the treatment of inflammatory disorders.

## Background

The Bixaceae family is one of the smallest plant families, consisting only of one genus, *Bixa*. There are only five species grouped under a single genus, and the most common species is *Bixa orellana*, an evergreen shrub grown not only because of its beautiful red flowers and ornamental red spiny fruits, but also for its economic value. *Bixa orellana*, also known as “annatto”, is native to tropical America [1], but widely cultivated and naturalized throughout the tropics, including Malaysia. *Bixa orellana* is well known for its coloring agent and medicinal value. The seeds are sources of food coloring and a dye called annatto. Besides that, they are also used as treatment for illnesses like gonorrhea and asthma and have been traditionally used as gargle for sore throats. The bark and root can be used to treat fever; the leaves are used as a cure for snakebites, jaundice, diabetes and hypertension, especially in Trinidad and Tobago, while also being used as a postpartum medicine in Malaysia [2]. The leaves of *Bixa orellana* have been reported to have antimicrobial [3], antifungal, antileishmanial [4], anticonvulsant, analgesic [5] and anti-inflammatory activities [6].

Inflammation limits the damage to the body cells after invasion by foreign organisms or mechanical injury, but if goes uncontrolled and untreated, it becomes a threat leading to other illnesses such as arthritis, cardiovascular disease, asthma, and cancer. Histamine is one of the most common inflammatory mediators; it causes symptoms of allergic reactions that mostly involve acute inflammation mediated by the H_1_ histamine receptor [7]. The histamine H_1_ receptor is mainly expressed in endothelial cells, smooth muscle cells and brain, and contributes to vasodilation, increase of vascular permeability [8] and pain [9] at the cellular level, while causing enhanced production of intracellular calcium (Ca^2+^) and nitric oxide (NO) [10] at the molecular level.

We have previously reported the ability of the aqueous extract of *Bixa orellana* (AEBO) in suppressing inflammation induced by carrageenan (data not published) and bradykinin [6]. This study aimed to investigate whether the anti-inflammatory activity of AEBO was caused by the blocking of histamine action, and to determine the involvement of two biochemical mediators, NO and vascular endothelial growth factor (VEGF), in the reduction of inflammation by AEBO.

## Methods

### Chemicals

The following chemicals and reagents were used: histamine, loratadine, Evans Blue (Sigma Chemical Co. Ltd, Selangor, Malaysia), nitrite/nitrate assay kit (Roche, Kuala Lumpur, Malaysia) and Murine VEGF ELISA kit (BioVision, USA).

### Plant material and extraction

*Bixa orellana* leaves were procured from around Universiti Putra Malaysia. Leaves were identified and voucher samples (NL16) were deposited at the Phytomedicine Herbarium, Institute of Bioscience, Universiti Putra Malaysia, Selangor. *B. orellana* leaves were dried in an oven at 60°C for three consecutive days and ground into fine powder. The aqueous extract was obtained by mixing the powder together with distilled water at ratio 1:20, and kept in a water bath (40°C) for 24 h, followed by filtration [6]. The filtrate was kept at −80°C and lyophilized using a freeze drier, yielding 8.5% (w/w). At the time of use, the lyophilized extract was dissolved in distilled water at the desired concentration.

### Experimental animals

Experiments were conducted using male *Sprague–Dawley* rats weighing 200–250 g, purchased from Northern RK Supplier, Malaysia and housed at the Animal House of the Faculty of Medicine and Health Sciences, Universiti Putra Malaysia. The animals were divided into groups of six individuals (n = 6) and kept in plastic cages at room temperature (23 ± 1°C) in a 12-h light:dark cycle, with free access to standard chow diet and water. The animals were acclimatized to the laboratory conditions for at least 1 week before the beginning of each experiment. All experiments complied with the current guidelines for the care and use of laboratory animals and were approved by the Animal Care and Use Committee (ACUC), Faculty of Medicine and Health Sciences, Universiti Putra Malaysia (ACUC NO. UPM/FPSKPADS/F01-00176).

### Gas chromatography – mass spectrometry analysis

The aqueous extract of *B. orellana* leaves (AEBO) was analyzed by gas chromatography–mass spectrometry (GC-MS-QP5050A-Shimadzu). A Zebron ZB-FFAP capillary column with length 30.0 m x diameter 0.25 mm and film thickness 0.25 μm was used. The column oven temperature was kept at 50°C for 3 min, then increased up to 250°C at a rate of 20°C/min and held at 250°C for 20 min. The carrier gas (helium) was introduced into the column at a flow rate of 1.0 mL/min. Injector and interface temperatures were set to 250°C each. AEBO (1 μL) was injected manually into the GC-MS in splitless mode. Compounds were identified by comparing their mass spectra with those in the NIST library and by comparing their retention times with those of authentic standards.

### Histamine-induced paw edema in rats

The effect of oral administration of AEBO was determined by injection of histamine in the subplantar region of the right hind paw, according to the method described by [11] and [12]. Briefly, rats (*n* = 6) were pretreated with AEBO at doses of 50 and 150 mg/kg, for 4 consecutive days, before being injected with 0.1 mL histamine (0.1%). Control animals received a similar volume of vehicle (0.1 mL). Loratadine (10 mg/kg), a histamine H_1_ antagonist, was used as the reference drug and administered 60 min before histamine injection on the fourth day. Edema was identified by the changes in paw volume, measured using a plethysmometer (Model 7140, Ugo Basile, Italy) before histamine administration and at 60, 120, 180 and 240 min after histamine injection.

### Histamine-induced intraperitoneal vascular permeability assay

To evaluate the changes of intraperitoneal vascular permeability induced by histamine, Evans blue dye was used as an indicator according to the method previously described by Whittle [13]. Rats were injected intravenously with 5 mL/kg of 1% Evans blue solution 60 min after the oral administration of AEBO, vehicle, or loratadine. Rats were killed 30 min after the intraperitoneal injection of histamine. The concentration of Evans blue in the peritoneal fluid was measured via spectrophotometry at 610 nm wavelength.

### Histamine-induced nitric oxide (NO) assay

NO was measured according to the method of Moshage et al. [14], using a commercially available Nitrite/Nitrate Colorimetric Kit (Roche). For this assay, 0.1 mL of 0.1% histamine was injected intraplantarly on the fourth day and the paw tissue obtained was deproteinized with 35% sulfosalicylic acid. The tissues were homogenized and centrifuged to obtain the supernatant. Nitrate (NO_3_^-^) was converted into nitrite (NO_2_^-^) after reacting with nitrate reductase. Total nitrite was measured using Griess reagent with 1:1 ratio and read at OD 540 nm. The sum of nitrite and nitrates obtained from this procedure represented the NO concentration in paw tissue (μM/g wet weight of tissue).

### Histamine-induced vascular endothelial growth factor (VEGF) assay

The concentration of VEGF in rat hind paw was determined using Murine VEGF ELISA Kit (BioVision). Analysis of VEGF was performed using standard ELISA methodology according to the kit’s manual. Briefly, paw tissue was excised after 6 h of histamine-induced inflammation. The tissues were homogenized in pre-chilled lysis buffer containing protease inhibitor. The homogenate was centrifuged and VEGF level was determined from the supernatant obtained. All assays were performed in triplicate and results were expressed in ng/mL protein.

### Statistical analysis

All data were reported as mean ± SEM; *n* represented the number of animals in each group. Statistical comparisons were made by ANOVA followed by Tukey’s multiple comparison test. *P* values < 0.05 were considered significant.

## Results

### Chemical constituents of AEBO

**Figure 1:**
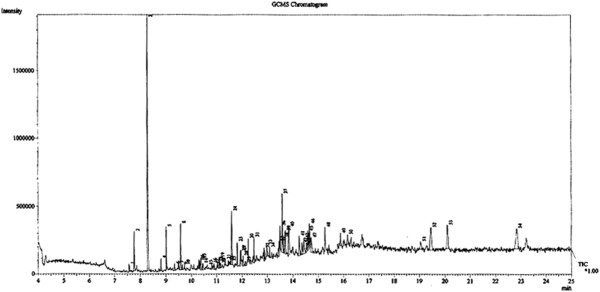
**Major chemical constituents present in the aqueous extract of *Bixa orellana *leaves.**

Several organic compounds were detected in AEBO by GC-MS (Figure 1). Six major compounds were identified: 2-butanamine (^t^R; 7.767 min), acetic acid (^t^R; 8.292 min), pentanoic acid (valeric acid) (^t^R; 9.583 min), phenol (^t^R; 11.600 min), pantolactone (^t^R; 11.817 min), and benzoic acid (^t^R; 13.850 min) (Table 1).

**Table 1:** **Chemical constituents of AEBO, detected by GC-MS, with relative retention time (**^**t**^**R)**

**Peak No.**	**Component**	**RT (min)**	**Peak Area (%)**	**Chemical family**
2	2-Butanamine	7.767	2.57	Amine
3	Acetic acid	8.292	19.95	Organic acid
8	Pentanoic acid	9.583	3.75	Fatty acid
24	Phenol	11.600	3.95	Carbolic acid
25	Pantolactone	11.817	1.53	Lactone
40	Benzoic acid	13.850	3.34	Organic acid

RT.

Retention.

Time.

### Effect of AEBO on histamine-induced paw edema in rats

**Figure 2:**
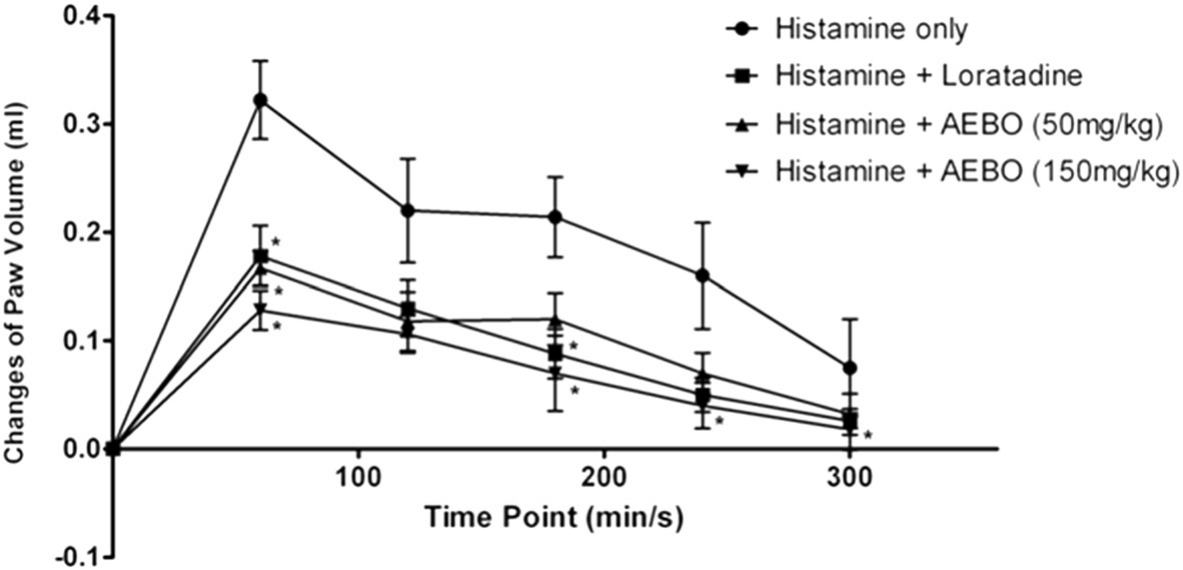
**Effect of AEBO on histamine-induced paw edema in rats. **Data are expressed as mean ± SEM, *n* = 6. Paw volume was measured every 60 min until 300 min. *P < 0.05, significantly different from negative control (histamine only).

Exposure of rat’s hind paw to histamine resulted in a marked increase of paw tissue weight and skin thickness. Paw weight significantly declined upon oral administration of AEBO (50 and 150 mg/kg) or loratadine (10 mg/kg) at 60 min time point compared with the non-treated group (Figure 2). Moreover, the changes were also significant for the dose of 150 mg/kg of AEBO compared to the non-treated group, from 180 min onwards. Although the paw volume reached its peak 60 min after histamine injection, the maximal edema inhibition was observed 300 min after the administration of 150 mg/kg of AEBO (76.0%). The percentages of inhibition produced by 150 mg/kg of AEBO at each time point (starting from 60 min) were 60.3%, 51.8%, 67.3%, 75.0% and 76.0%.

### Effect of AEBO on histamine-induced peritoneal vascular permeability

**Figure 3:**
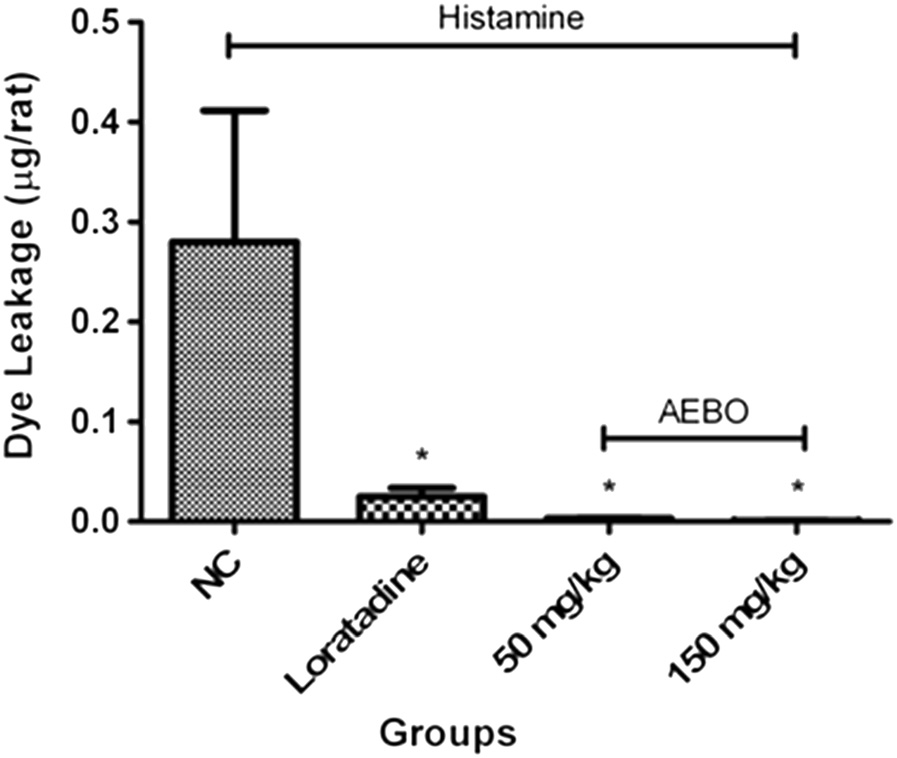
**Effect of AEBO on histamine-induced peritoneal vascular permeability. **Evans blue dye was collected and measured by spectrophotometer. Data are presented as mean ± SEM, *n* = 6. *P < 0.05, significantly different from NC (negative control).

As shown in Figure 3, both doses of AEBO (50 and 150 mg/kg) notably inhibited the histamine-induced increase of dye leakage into the peritoneal cavity, with an inhibitory rate up to 99%. The potency of AEBO was similar to that of loratadine (91.1% inhibition).

### Effect of AEBO on histamine-induced NO production

**Figure 4:**
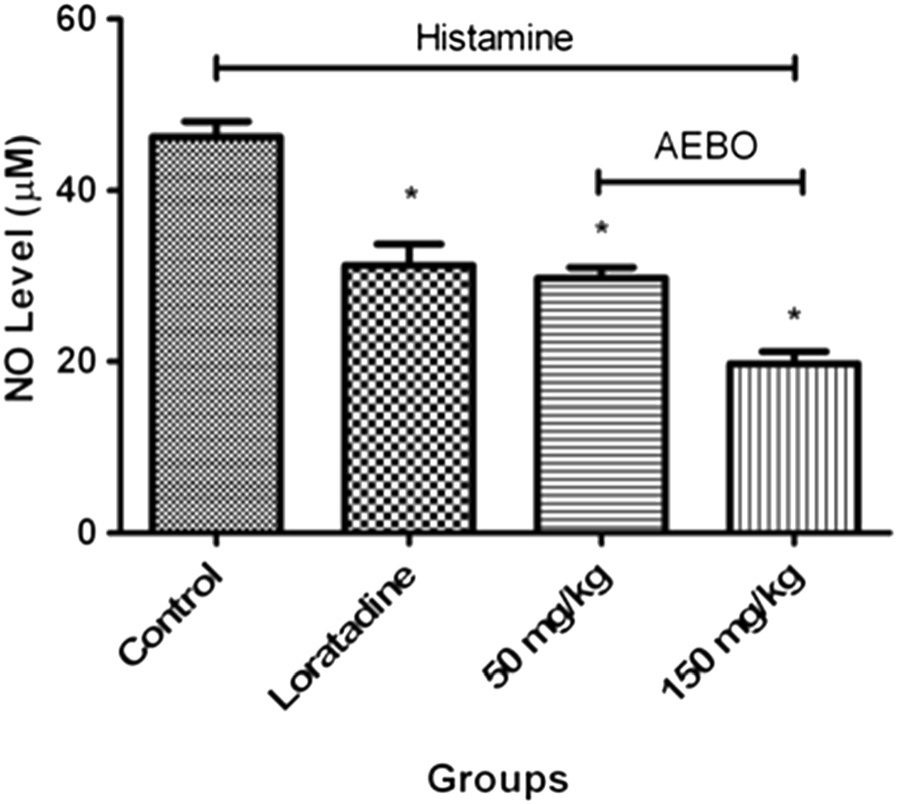
**Effect of AEBO on histamine-induced NO production in rat’s paw. **Paw tissue was homogenized and the supernatant was assayed by the Griess reaction. Data are presented as mean ± SEM, *n* = 6. *P < 0.05, significantly different from control.

Production of NO in rat’s paw was markedly increased in response to histamine (46.2 ± 1.8 μM), but was significantly suppressed by both doses of AEBO (*P* < 0.05) (Figure 4). The concentrations of NO were reduced to 29.7 ± 1.2 μM and 19.7 ± 1.4 μM after the administration of 50 and 150 mg/kg of AEBO, respectively. On the other hand, loratadine, the reference drug, also significantly inhibited the NO production induced by histamine. However, the inhibition rate of loratadine was not as high as that obtained with both doses of AEBO (50 and 150 mg/kg). This indicated that AEBO had an inhibitory effect on histamine-induced NO production.

### Effect of AEBO on histamine-induced VEGF production

**Figure 5:**
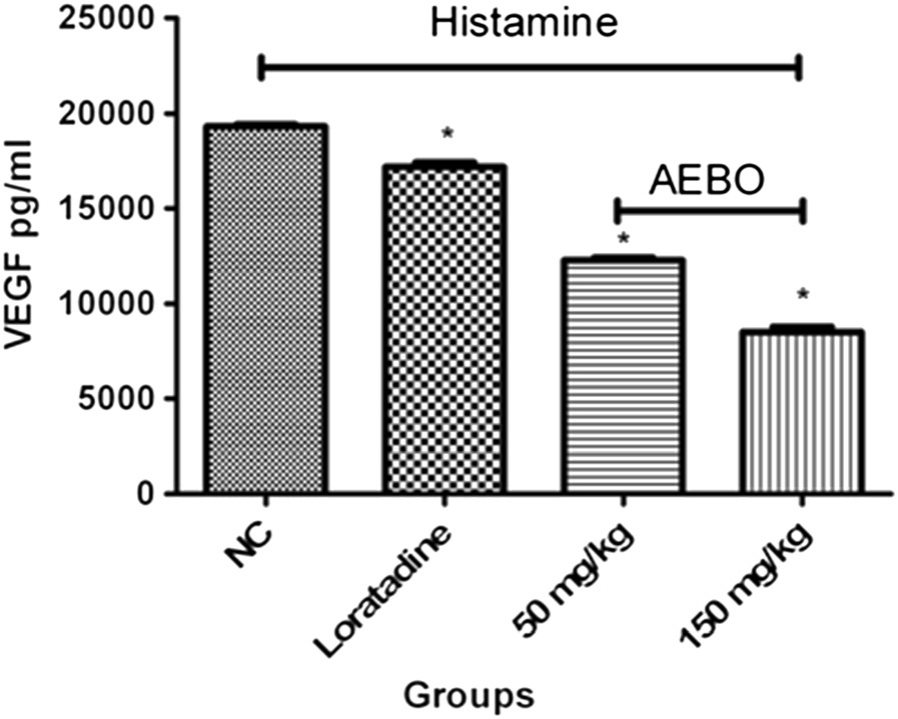
**Influence of AEBO on histamine-induced VEGF production in rat’s paw. **Data are presented as mean ± SEM, *n* = 6. *P < 0.05, significantly different from NC (negative control).

Figure 5 shows that oral administration of AEBO significantly suppressed histamine-induced VEGF production in rat’s paw. As observed, the highest dose of AEBO (150 mg/kg) had a greater effect (55.9% inhibition) compared with the reference drug, loratadine (~11.2% inhibition). On the other hand, 50 mg/kg of AEBO only showed 36.6% inhibition compared with the non-treated group.

## Discussion

The present study demonstrated that the aqueous extract of *Bixa orellana* leaves had anti-inflammatory activity, as shown by the suppression of vascular fluid extravasations produced by histamine. These antihistamine activities, revealed by decreased paw volumes and almost normalized peritoneal vascular permeability, were suspected to be aided by the suppression of other permeability-regulating substances (NO and VEGF).

In our preliminary study, *B.orellana* exhibited the ability to suppress the inflammatory activities of a number of mediators, with AEBO having inhibited up to 99% of paw edema induced by carrageenan (data not published). Indeed, when bradykinin was later used to induce acute inflammation in the same rat models, it was confirmed that up to 82% of the increased vascular permeability was successfully alleviated upon administration of AEBO [6]. Histamine is one of the inflammogens that contributes to acute inflammation and increase of vascular permeability [15]. The present study documents for the first time the ability of AEBO to suppress the histamine-induced inflammation in rats. Paw edema assay is a useful tool for investigating agents with potential anti-inflammatory capabilities. The present study showed that exposure of rat’s paw to histamine triggered an elevation in fluid extravasations from microvessels in the vicinity, an event that led to tissue swelling. The results showed that AEBO was able to significantly suppress histamine-induced paw edema in rats, and this may probably be due to the inhibition of the H_1_ receptor or other signaling molecules down its pathway. Histamine H_1_ receptor expressed in endothelial cells of blood vessels is important for regulating vascular permeability [10], and increased permeability occurs once it is activated.

Inflammatory processes are also characterized by an increase in microvascular permeability to macromolecules. Leakage of Evans blue dye into the peritoneal cavity indicated the formation of gaps or pores in the wall of the peritoneal microvessels, large enough for a significant portion of macromolecules to pass through. The administration of AEBO resulted in the retention of the normal endothelial barrier function during histamine challenge, having prevented the leakage of approximately 99% of the Evans blue dye into the peritoneal cavity. Based on these outcomes, it was demonstrated that AEBO displayed significant antihistamine activity, through inhibition of the vascular permeability processes. This could also be related to the suppression of other permeability-regulating molecules that lead to inflammation.

Vascular endothelial growth factors (VEGFs) are known as key regulators of permeability and binding to their receptors trigger an increase in vascular permeability [16]. Upregulation and downregulation of VEGF expression contributes to abnormal angiogenesis indirectly leading to vascular hyperpermeability [17]. Furthermore, overproduction of VEGF also contributes to progression to other diseases such as coronary disease [18] and cancer [19]. Ghosh and colleagues [20] demonstrated that histamine enhanced the production of VEGF during acute inflammation. VEGF release and its above-mentioned effects on vascular permeability and cellular infiltration could contribute to edema formation. Thus, it is very important to regulate VEGF production in cells either by suppression when it is overproduced or vice versa. Our results showed that the oral administration of AEBO in rats decreased the histamine-induced VEGF production, indicating that AEBO may have the potential to regulate vascular permeability by monitoring the expression of VEGF.

Nitric oxide (NO) is a signaling molecule that is synthesized from L-arginine by NO synthase [21]. It is well recognized that NO plays an important role in maintaining the normal physiological function of vascular tissues. It has been previously reported that histamine increased vascular permeability by activating NO production [22]. In addition, Mayhan [23] also demonstrated that nitric oxide was involved in increased macromolecular extravasation induced by histamine in the hamster cheek pouch. In addition, endothelial nitric oxide synthase (eNOS) was found to play a major role in vascular leakage during acute inflammation *in vivo*[24]. Thus, suppression of NO production may help reduce acute inflammation. From the present study, it was observed that more than 50% of the NO elevation caused by histamine could be alleviated by AEBO. This further supports its action as an anti-inflammatory agent.

Raga and colleagues [25] successfully isolated ishwarane, phytol, polyprenol and a mixture of stigmasterol and sitosterol from the dichloromethane extract of air-dried leaves of *B. orellana*. The current GC-MS analysis revealed six known chemical constituents. However, based on the literature, only four of them are known to exhibit pharmacological activity. Acetic acid is an organic acid that has been reported to possess antifungal [26] and antibacterial properties [27]. Pentanoic acid (valeric acid) is a sedative, anticonvulsant and mood-stabilizing agent [28]; whereas phenol was reported to have anesthetic/analgesic [29] and antiseptic [30] activities. Benzoic acid possesses antibacterial [31] and antifungal properties [32]. As for their anti-inflammatory contribution, a low concentration of acetic acid (0.3 mg/mL) has been shown to inhibit histamine release from guinea pig lung mast cells when stimulated by both antigen (ovalbumin) and ionophore A23187 [33]. It is known that allergic reactions develop from large inflammatory responses caused by the release of mediators, including histamine, during degranulation of mast cells. There is also evidence of histamine itself acting as an inducer of mast cell degranulation [34]. This could have happened in our rat model, where histamine was injected into animals to induce inflammation.

All of these constituents may or may not play an important role in the anti-inflammatory activity. Nonetheless, the possibility of the minor compounds exhibiting anti-inflammatory properties cannot be excluded.

## Conclusions

Our results confirmed that the aqueous extract of *B. orellana* had antihistamine activities, as demonstrated by the suppression of increased vascular permeability reflected in the decreased paw volume and Evans blue dye leakage. It appeared that this permeability modulation by AEBO also involved the reduction of biochemical mediators such as NO and VEGF. We considered that the active principle contained in the aqueous extract may play a role in its anti-inflammatory activity. Whether or not it acts by stabilizing mast cells remains to be elucidated.

## Competing interests

The authors declare that they have no competing interests.

## Authors’ contributions

YYK, ZAZ, AAK, MNS, GECL and ZA participated in the design of the research. YYK carried out the experiments, analyzed the data and wrote the paper. ZA provided funding and supervised the study. All authors read and approved the final manuscript.

## Pre-publication history

The pre-publication history for this paper can be accessed here:

http://www.biomedcentral.com/1472-6882/13/32/prepub
